# Comprehensive Geriatric Assessment and Nutrition-Related Assessment: A Cross-Sectional Survey for Health Professionals

**DOI:** 10.3390/geriatrics4010023

**Published:** 2019-02-15

**Authors:** Junko Ueshima, Keisuke Maeda, Hidetaka Wakabayashi, Shinta Nishioka, Saori Nakahara, Yoji Kokura

**Affiliations:** 1 Department of Clinical Nutrition and Food Service, NTT Medical Center Tokyo, Tokyo 141-0022, Japan; 2 Palliative Care Center, Aichi Medical University, Aichi 480-1195, Japan; kskmaeda@aichi-med-u.ac.jp; 3 Department of Rehabilitation Medicine, Yokohama City University Medical Center, Yokohama 232-0024, Japan; noventurenoglory@gmail.com; 4 Department of Clinical Nutrition and Food Services, Nagasaki Rehabilitation Hospital, Nagasaki 850-0854, Japan; shintacks@yahoo.co.jp; 5 Department of Nutrition, Suzuka General Hospital, Suzuka 513-8630, Japan; mikuhiroto1210@gmail.com; 6 Department of Clinical Nutrition, Keiju Medical Center, Nanao 926-8605, Japan; yojikokura@hotmail.com

**Keywords:** comprehensive geriatric assessment, multicomponent assessment, rehabilitation nutrition, sarcopenia, sarcopenic dysphagia

## Abstract

(1) Background: It is important to assess physical and nutritional status using the Comprehensive Geriatric Assessment (CGA). However, the correlation between the CGA usage and nutritional-related assessments remain unclear. This study aims to clarify the correlation between the CGA usage and other nutritional-related assessments. (2) Methods: We conducted a questionnaire survey on clinical use of CGA, assessment of sarcopenia/sarcopenic dysphagia/cachexia, and defining nutritional goals/the Nutrition Care Process/the International Classification of Functioning, Disability, and Health (ICF)/the Kuchi–Kara Taberu Index. (3) Results: The number of respondents was 652 (response rate, 12.0%), including 77 who used the CGA in the general practice. The univariate analyses revealed that participants using the CGA tended to assess sarcopenia (*P* = 0.029), sarcopenic dysphagia (*P* = 0.001), and define nutritional goals (*P* < 0.001). Multivariate logistic regression analyses for the CGA usage revealed that using ICF (*P* < 0.001), assessing sarcopenia (*P* = 0.001), sarcopenic dysphagia (*P* = 0.022), and cachexia (*P* = 0.039), and defining nutritional goals (*P* = 0.001) were statistically significant after adjusting for confounders. (4) Conclusions: There are correlations between the use of CGA and evaluation of sarcopenia, sarcopenic dysphagia, and cachexia and nutritional goals.

## 1. Introduction

The rapidly aging society warrants continuous advancements of the conventional medical care [1,2]. As frail older adults should have access to comprehensive medical and nursing care, provision of comprehensive care using multicomponent assessment is imperative [2]. The Comprehensive Geriatric Assessment (CGA) is a multidimensional, interdisciplinary diagnostic and treatment process that is designed to collect data about medical aspects of frail older adults [3,4]. The primary components of various models of the CGA comprise the coordinated multidisciplinary assessment, geriatric medicine expertise, determining medical, physical, social, and psychological problems, and the creation of a care plan involving appropriate rehabilitation [4]. Compared to typical medical care, the CGA implementation enhances the survival time of older adults, increases the duration during which they can live at home, and, perhaps, improves cognitive functions [1,4], improving their quality of life (QOL). Despite being recommended to be used in in the clinical practice, the CGA remains only partially utilized [5].

Sarcopenia, a key contributor of frailty [6], is a syndrome characterized by the presence of both the muscle mass and muscle function reduction due to aging, inactivity, malnutrition, and conditions such as cachexia [7]. Reportedly, sarcopenia is associated with an increased mortality and healthcare costs and declined QOL [8], and is considered as a severe public health-related concern [8,9]. Recently, some studies have described sarcopenic dysphagia (dysphagia due to sarcopenia in the whole body and swallowing-related muscles.) [10,11,12,13,14], which is occasionally detected in older adults and is related to physical deterioration, inadequate nutrition management, and cognitive decline [15]. Perhaps multicomponent assessment, such as the assessment of physical, social, and psychological problems, appropriate rehabilitation, and nutrition management could be necessary for the treatment [14,15], necessitating the early diagnosis of sarcopenia. In Nakahara et al. [16], we clarified the evaluation of sarcopenia and cachexia among different occupations, but when these items were evaluated remains unclear. In the rapidly aging society of Japan, the number of older adults with sarcopenia, nutritional deficiency, weakness, and disability are increasing at an alarming rate [17]. Therefore, it is important to assess physical functions and nutritional status as well as using CGA and to extract patients at risk early.

The usage of the multicomponent assessment is desirable to evaluate the elderly clinically and has been projected to attain a shared understanding of assessment and intervention goals. Besides the CGA, there are other multicomponent tools that clinically assess older adults such as the International Classification of Functioning, Disability, and Health (ICF) and the Kuchi–Kara Taberu Index (KT index) [18]. Wakabayashi et al. [11] advocates care that can maximize the physical function, physical activity, and social participation by assessing patients by ICF, including the nutritional status. Maeda et al. [18] recommended the multicomponent assessment and nutrition management for patients with eating and swallowing problems using the KT index. Recently, ICF-Dietetics [19] has been established as a systematically problem-solving method for ICF-related nutritional issues. The CGA usage mandates defining nutritional goals and controlling nutrition using nutritional problem-solving methods, such as the Nutrition Care Process (NCP) [20] and nutrition management, with a common understanding among healthcare workers in different occupations. Based on these, Wakabayashi [17] suggested providing high-quality nutritional care using the rehabilitation nutrition care process to people with disability and frailty. The rehabilitation nutrition care process assesses frailty, sarcopenia, dysphagia, and cachexia after using multicomponent assessment tools such as ICF or CGA and KT index. However, the rehabilitation nutrition care process has just begun, and the correlation between the CGA usage and the assessment of sarcopenia, cachexia, and sarcopenic dysphagia and defining nutritional goals using the NCP remain unclear. Moreover, the correlation between the CGA usage and the ICF usage and the KT index remains unknown.

In addition, a medical fee has been obtained since 2008 owing to the implementation of the CGA in Japan. Kihon Checklist [21] is an example of the CGA; although the assessment of frailty and assessment of muscle strength and physical functions are included in its components, the assessment of muscle mass and cachexia is not included.

Therefore, this study aims to elucidate the implementation rate and correlation between using the CGA and using nutrition-related assessment items, such as assessment of sarcopenia, cachexia, and sarcopenic dysphagia, NCP, defining nutritional goals, through a questionnaire-based survey. It also explains the implementation rate of CGA among different types of healthcare professionals and settings. Furthermore, this study intends to assess the correlation between the CGA usage and the use of other multicomponent tools, including the ICF and KT index.

## 2. Materials and Methods

### 2.1. Study Design and Setting

Between December 9, 2016, and January 16, 2017, we conducted a cross-sectional study using questionnaires. The questionnaire respondents were members of the Japanese Association of Rehabilitation Nutrition, which was established in 2011 and includes 5520 members from various medical and healthcare specialties. We conducted the survey online and anonymously to protect respondents’ personal information and guarantee confidentiality. 

### 2.2. Ethical Considerations

This study was performed following the ethical guidelines of the Declaration of Helsinki and was approved by the Ethics Committee of Suzuka General Hospital at Mie prefecture in Japan (No. 161). With answers to the questionnaire, we explained to the participants that they consented to the research and were given responses. For the protection of personal information in completed questionnaires, full confidentiality was given to respondents’ data.

### 2.3. Participants

In this study, we enrolled respondents who provided consent to participate in the survey and responded to the questionnaire. Of note, those with missing data or duplications were excluded from the analysis.

### 2.4. Data Collection

We conducted the survey online, and it took approximately 5 min to complete the online questionnaire that comprised selective questions with dichotomous choice (yes/no). The consistency of the questionnaire content was evaluated by researchers. After several investigators conducted preliminary tests, the questionnaire content was enhanced regarding phrases, forms, length, consistency, and ease of answering, followed by converting into an actual survey. Table A1 lists the questions asked in the survey.

### 2.5. Parameters

In this study, we assessed parameters such as the standard implementation of the CGA, ICF, KT index, sarcopenia, sarcopenic dysphagia, cachexia, defining nutritional intervention goals, and the usage of NCP. The characterization of each evaluation item is in the Appendix (Table A2).

### 2.6. Statistical Analysis

Data analysis was performed on a sample size of >664 respondents, under the assumption of two-choice questions, and 50% selection with a 5% error based on 99% reliability. We expressed all categorical variables as the number of individuals and percentages. In addition, we performed a comparison of groups using the *χ*^2^ test.

All quantitative variables were expressed as median (interquartile ranges). We used the Mann–Whitney *U*-test to compare values of the length of work experience. In addition, univariate and multivariate logistic regression analyses were performed to estimate the adjusted odds ratios (OR). Of note, the confounders were occupations, affiliations, and length of work experience. We performed all statistical procedures using EZR [22] software version 1.31, which was developed from the open-source statistical software R [23]. Furthermore, we considered *P* < 0.05 as statistically significant.

## 3. Results

Of 660 respondents, we excluded 8 (1.2%) from analyses because of incomprehensible answers. Consequently, the number of valid respondents was 652 (response rate, 12.0%). Table 1 summarizes the characteristics of the study cohort. The leading occupation of respondents was registered dietitian (28.2%), followed by a physical therapist (26.4%) and speech therapist (15.5%). Affiliations were with acute care (37.9%), convalescent rehabilitation (26.5%), nursing homes (10.4%), home care service (10.3%), long-term care (6.9%), and others (8%). Besides, the median work experience was 12 (range, 7–18) years.

Overall, 77 (11.8%) respondents were using the CGA in the general practice. The univariate analysis revealed that people using the CGA were more likely to assess sarcopenia (*P* = 0.029), sarcopenic dysphagia (*P* = 0.001), and define nutritional goals (*P* < 0.001). In contrast, using the ICF (*P* = 0.051), KT index (*P* = 0.120), and NCP (*P* = 0.144) and assessing cachexia (*P* = 0.054) was not significantly different (see Table 2).

The logistic regression analyses established a correlation between the CGA usage and several factors (see Table 3); these factors included using the ICF (adjusted OR, 3.01; 95% confidence interval [CI]: 1.63–3.57; *P* < 0.001), assessing sarcopenia (adjusted OR, 2.60; 95% CI: 1.50–4.50; *P* = 0.001), assessing sarcopenic dysphagia (adjusted OR, 1.86; 95% CI: 1.09–3.16; *P* = 0.022), assessing cachexia (adjusted OR, 1.86; 95% CI: 1.03–3.34; *P* = 0.039), and setting nutritional goals (adjusted OR, 2.79; 95% CI: 1.56–4.98; *P* = 0.001), which we observed with a statistical significance. Furthermore, the use of the KT index and NCP did not correlate with the CGA use.

## 4. Discussion

In brief, this study revealed three significant findings. First, it was suggested that participants using the CGA may have assessed sarcopenia or sarcopenic dysphagia more frequently in the daily clinical practice than those not using the CGA. Second, participants using the CGA defined nutritional goals more frequently; however, no significant difference was observed in using the NCP. Third, the percentage of people using the CGA was as low as 11.8%.

Participants using the CGA may have assessed sarcopenia or sarcopenic dysphagia more frequently in the daily clinical practice than those not using the CGA. Although there have been few studies on the relevance of assessing the CGA and sarcopenia. In recent studies, assessment of frailty was included as a component of CGA, but there was no sarcopenia [24]. Kihon Checklist [21] is a sample implementation of the CGA in Japan. It includes assessment of frailty, muscle strength, and physical functions in its components, and it has one aspect that motivates the assessment of sarcopenia. Sarcopenia is not only related to the physical activity and dysfunction [25,26] but also with several other factors, including independence [27,28] and cognitive function [29] of the daily life. In addition, sarcopenic dysphagia correlates with nutrition, activity (physical activity and swallowing), and cognitive function, which are causal factors of secondary sarcopenia [15]. Reportedly, the prevalence of sarcopenia among older adults is 1–29% in community dwellers, 14–33% in long-term care facilities, and 10–35% in acute-care hospitals [30,31]. Furthermore, it is a factor that predicts the life expectancy and disability. In fact, it is imperative to screen older adults who are susceptible to sarcopenia and sarcopenic dysphagia in the CGA and intervene at an early stage. As the CGA is the accepted gold standard for caring for frail older people in hospitals [24], it is essential to assess sarcopenia and sarcopenic dysphagia as a prolongation of the nutritional assessment and physical function evaluation of the CGA.

Participants using the CGA define nutritional goals more frequently; however, we observed no statistically significant difference in using the NCP. DiMaria-Ghalili et al. [32] reported that because all regions of the CGA and nutritional status were related, the nutritional assessment in the CGA facilitated the early recognition of nutritional risk factors or malnutrition, raising the possibility of a timely intervention. A study reported that it is crucial to illustrate the setting of nutritional goals at the time of the intervention after the nutrition assessment [33]. Although defining nutrition goals is encouraged for the NCP, a method of systematically solving nutrition-related problems, we observed no significant differences in using the NCP. Perhaps, participants related the nutritional assessment to the CGA, but the nutrition goal setting was implemented by methods other than the NCP. In Japan, NCP education has been initiated only recently for registered dietitians, and the NCP has not yet been applied in several occupations. In future, it will be crucial to define nutritional goals using nutrition-related problem-solving methods that could be shared among multiple occupations in Japan.

Among our study participants, the implementation rate of the CGA was as low as 11.8%, which was particularly low in acute-care hospitals. Apparently, the interdisciplinary work is necessary for the CGA implementation. We conjecture that the emergency departments of acute-care hospitals prioritize professional care over the CGA [5], or such an interdisciplinary working model has not been established [34]. Gladman et al. [5] reported that the CGA is challenging to comprehend and implement, even among those who care for the elderly. Li et al. [34] reported that even when the CGA was indicated, its implementation rate was as low as 20% because of inadequate education. In Japan, CGA education exists for medical doctors and dentists provided by gerontologists, but such an education is not provided for other medical professionals, although it is essential that all healthcare professionals should have access to adequate education. We need to clarify the reason why we are assessing sarcopenia and not using CGA, although we could not describe in this research. Thereby, we believe that the issue at the clinical practice will be highlighted.

This study has several limitations. First, based on the questionnaire response rate of only 12.0%, it is difficult to generalize our findings to the entire country. Second, it remains unclear how the questionnaire respondents diagnosed sarcopenia, sarcopenic dysphagia, and cachexia. When conducting similar research next time, it is necessary to describe diagnostic criteria of sarcopenia, sarcopenic dysphagia, and cachexia. If we clarify the reason why participants assess sarcopenia, sarcopenic dysphagia, cachexia, and the CGA, we can get more insights for clinical practice.

## 5. Conclusions

In conclusion, this study establishes correlations between the CGA usage and evaluation of sarcopenia, sarcopenic dysphagia, and cachexia and nutritional goals. In addition, those using the CGA are highly likely to assess older adults with a more multidimensional approach. However, the presence of few implementers is problematic. It is essential to extract older adults susceptible to sarcopenia at an early stage with an appropriate care plan, including the rehabilitation and nutrition management. Therefore, in the future it will be necessary to include items for the evaluation of sarcopenia, sarcopenic dysphagia, and cachexia in the CGA.

## Figures and Tables

**Table 1:** Demographic Characteristics of Participants.

Characteristics, n (%)	All	Usage of Comprehensive Geriatric Assessment		*P*-Value
		No	Yes	
Total Occupation	652 (100)	575 (88.2)	77 (11.8)	
Registered dietitian	184 (28.2)	162 (88.0)	22 (12.0)	0.01 ^a^
Physical therapist	172 (26.4)	156 (90.7)	16 (9.3)	
Speech therapist	101 (15.5)	93 (92.1)	8 (7.9)	
Nurse	60 (9.2)	54 (90.0)	6 (10.0)	
Medical doctor	43 (6.6)	32 (74.4)	11 (25.6)	
Occupational therapist	36 (5.5)	33 (91.7)	3 (8.3)	
Dental hygienist	24 (3.7)	22 (91.7)	2 (8.3)	
Dentist	24 (3.7)	18 (75.0)	6 (25.0)	
Pharmacist	7 (1.1)	4 (57.1)	3 (42.9)	
Certified care worker	1 (0.1)	1 (100.0)	0 (0.0)	
Affiliation				
Acute care	247 (37.9)	231 (40.2)	16 (20.8)	0.02 ^a^
Convalescent rehabilitation	173 (26.5)	149 (25.9)	24 (31.2)	
Nursing home	68 (10.4)	59 (10.3)	9 (11.7)	
Homecare service	67 (10.3)	53 (9.2)	14 (18.2)	
Medical care or long-term care	45 (6.9)	39 (6.8)	6 (7.8)	
Others	52 (8.0)	44 (7.7)	8 (10.4)	
Work experience				
Year(s), median (25–75%)	12 (7–18)	12 (7–18)	11(7–20)	0.85 ^b^

^a^ Chi-square test; ^b^ Mann–Whitney *U*-test.

**Table 2:** Univariate Analysis of Factors Associated with Usage of Comprehensive Geriatric Assessment.

Factor, n (%)	All	Usage of Comprehensive Geriatric Assessment		*P*-Value
		No	Yes	
Using the ICF				
No	289 (44.3)	263 (45.7)	26 (33.8)	0.05
Yes	363 (55.7)	312 (54.3)	51 (66.2)	
Using the KT index				
No	557 (85.4)	496 (86.3)	61 (79.2)	0.12
Yes	95 (14.6)	79 (13.7)	16 (20.8)	
Assessing sarcopenia				
No	315 (48.3)	287 (49.9)	28 (36.4)	0.03
Yes	337 (51.7)	288 (50.1)	49 (63.6)	
Assessing sarcopenic dysphagia				
No	432 (66.3)	394 (68.5)	38 (49.4)	0.001
Yes	220 (33.7)	181 (31.5)	39 (50.6)	
Assessing cachexia				
No	478 (73.3)	429 (74.6)	49 (63.6)	0.05
Yes	174 (26.7)	146 (25.4)	28 (36.4)	
Setting nutritional goal				
No	359 (55.1)	333 (57.9)	26 (33.8)	<0.001
Yes	293 (44.9)	242 (42.1)	51 (66.2)	
Using Nutrition Care Process				
No	569 (87.3)	506 (88.0)	63 (81.8)	0.14
Yes	83 (12.7)	69 (12.0)	14 (18.2)	

Abbreviations: ICF, International Classification of Functioning, Disability and Health; KT index, Kuchi–Kara Taberu Index.

**Table 3:** Odds Ratio of Comprehensive Geriatric Assessment Usage in Uni- and Multi-Variate Logistic Regression Analyses.

Dependent Variables	Usage of Comprehensive Geriatric Assessment					
	Unadjusted OR	95% CI	*P*-Value	Adjusted OR	95% CI	*P*-Value
Using the ICF	1.65	0.98–2.84	0.05	3.01	1.63–5.57	<0.001
Using the KT index	1.66	0.84–3.07	0.12	1.76	0.91–3.43	0.10
Assessing sarcopenia	1.74	1.04–2.97	0.03	2.60	1.50–4.50	0.02
Assessing sarcopenic dysphagia	2.23	1.34–3.72	0.001	1.86	1.09–3.16	0.001
Assessing cachexia	1.68	0.98–2.84	0.05	1.86	1.03–3.34	0.04
Setting nutritional goal	2.70	1.60–4.64	<0.001	2.79	1.56–4.98	0.001
Using Nutrition Care Process	1.63	0.80–3.14	0.14	1.59	0.75–3.37	0.23

For each multivariate regression model, usage of CGA was adjusted by occupations, affiliations, and length of work experience. Abbreviations: ICF, International Classification of Functioning, Disability and Health; KT index, Kuchi–Kara Taberu Index; OR, odds ratio; 95% CI, 95% confidence interval.

## Appendix A

**Table A1:** Questionnaire contents.

Questions	Options
Q1. What is your occupation?	Registered dietitian
	Physical therapist
	Speech therapist
	Nurse
	Medical doctor
	Occupational therapist
	Dental hygienist
	Dentist
	Pharmacist
	Certified Care Worker
Q2. What is your sex?	Male
	Female
Q3. What is your affiliation?	Acute care
	Recovery rehabilitation
	Long-term care health facility
	Homecare service
	Medical care or long-term care
	Others
Q4. How long is your work experience?	
Q5. Do you use the CGA?	Yes/No
Q6. Do you assess sarcopenia?	Yes/No
Q7. Do you assess sarcopenic dysphagia?	Yes/No
Q8. Do you assess cachexia?	Yes/No
Q9. Do you set nutritional goals?	Yes/No
Q10. Do you use KT index?	Yes/No
Q11. Do you use the Nutrition Care Process?	Yes/No
Q12. Do you use the ICF?	Yes/No

Abbreviations: CGA, Comprehensive geriatric assessment; KT index, Kuchi–Kara Taberu Index; ICF, International Classification of Functioning, Disability and Health.

**Table A2:** The characterization of each evaluation items.

The ICF	The WHO framework for measuring health and disability at both individual and population levels. It is classified according to a combination of alphabet and number, and it consists of three factors, “physical and mental function/physical structure,” “activity” and “participation,” and influential factors, such as “environment” and “individual” [35].
The KT index	A simplified, validated tool that comprehensively assesses and intervenes in problems associated with eating and swallowing. The index comprises the following 13 items: (1) willingness to eat, (2) overall condition, (3) respiratory condition, (4) oral condition, (5) cognitive function while eating, (6) oral preparatory and propulsive phases, (7) severity of pharyngeal dysphagia, (8) position and endurance while eating, (9) eating behavior, (10) daily living activities, (11) food intake level, (12) food modification, and (13) nutritional status [18]. As each item is rated from 1 (worst) to 5 (best) points, the KT index ranges from 13 to 65 and is drawn with a radar chart that facilitates determining strong and weak items to ascertain items that caregivers need to emphasize and recognize the effect of an intervention by comparing before and after results.
The NCP	A systematic approach to provide high-quality nutritional care to patients/clients and is the unique function of nutrition in a standardized language through four related steps as follows: (1) nutrition assessment, (2) nutrition diagnosis, (3) nutrition management, and (4) nutrition monitoring and evaluation [20].

Abbreviations: WHO, World Health Organization; KT index, Kuchi–Kara Taberu Index; ICF, International Classification of Functioning, Disability and Health; NCP, Nutrition Care Process.
